# Rhein attenuates angiotensin II-induced cardiac remodeling by modulating AMPK–FGF23 signaling

**DOI:** 10.1186/s12967-022-03482-9

**Published:** 2022-07-06

**Authors:** Wei Lu, Hongqiao Zhu, Jiawen Wu, Sheng Liao, Guobing Cheng, Xiaoyang Li

**Affiliations:** 1 Department of Cardiovascular Surgery, The Quzhou Affiliated Hospital of Wenzhou Medical University, Quzhou People’s Hospital, Quzhou, 324000 China; 2 Department of Vascular Surgery, Changhai Hospital, Navy Medical University, Shanghai, 200433 China

**Keywords:** Rhein, Cardiac remodeling, ROS, AMPK, FGF23

## Abstract

**Background:**

Increasing evidence indicates that myocardial oxidative injury plays a crucial role in the pathophysiology of cardiac hypertrophy (CH) and heart failure (HF). The active component of rhubarb, rhein exerts significant actions on oxidative stress and inflammation. Nonetheless, its role in cardiac remodeling remains unclear.

**Methods:**

CH was induced by angiotensin II (Ang II, 1.4 mg/kg/d for 4 weeks) in male C57BL/6 J mice. Then, rhein (50 and 100 mg/kg) was injected intraperitoneally for 28 days. CH, fibrosis, oxidative stress, and cardiac function in the mice were examined. In vitro, neonatal rat cardiomyocytes (CMs) and cardiac fibroblasts (CFs) pre-treated with rhein (5 and 25 μM) were challenged with Ang II. We performed RNA sequencing to determine the mechanistic role of rhein in the heart.

**Results:**

Rhein significantly suppressed Ang II-induced CH, fibrosis, and reactive oxygen species production and improved cardiac systolic dysfunction in vivo. In vitro, rhein significantly attenuated Ang II-induced CM hypertrophy and CF collagen expression. In addition, rhein obviously alleviated the increased production of superoxide induced by Ang II. Mechanistically, rhein inhibited FGF23 expression significantly. Furthermore, *FGF23* overexpression abolished the protective effects of rhein on CMs, CFs, and cardiac remodeling. Rhein reduced *FGF23* expression, mostly through the activation of AMPK (AMP-activated protein kinase). AMPK activity inhibition suppressed Ang II-induced CM hypertrophy and CF phenotypic transformation.

**Conclusion:**

Rhein inhibited Ang II-induced CH, fibrosis, and oxidative stress during cardiac remodeling through the AMPK–FGF23 axis. These findings suggested that rhein could serve as a potential therapy in cardiac remodeling and HF.

**Supplementary Information:**

The online version contains supplementary material available at 10.1186/s12967-022-03482-9.

## Introduction

Hypertensive cardiac disease features a hypertrophic left ventricle and fibrotic interstitium, which ultimately leads to heart failure (HF) [1]. These prior alterations are initially adaptive reactions for sustaining ventricular functionality, but persistent hypertrophy is detrimental [2]. There is increasing evidence that the progression of cardiac hypertrophy (CH) to dilated cardiomyopathy (DCM) is inevitable and which eventually progresses to HF [3–5]. Therefore, suppressing hypertensive CH may impede progression to HF. Although the clinical management of hypertensive heart disease has been explored in multiple dimensions, there remain few feasible medications for preventing or even reversing CH progression [6].

Clinical and experimental studies have reported the pivotal effect exerted by the renin–angiotensin–aldosterone system in diverse cardiovascular conditions [7]. As the primary effector of the renin-angiotensin system, angiotensin II (Ang II) is tightly linked to autophagic incompetence, oxidative stress, and the inflammatory response [8]. Ang II initiates several signaling axes, such as that of MAPK (mitogen-activated protein kinases), AMP-activated protein kinase (AMPK), and NF-κB, to induce cardiac remodeling [4, 9]. Furthermore, it has been demonstrated that preventing myocardial hypertrophy and fibrosis is possible by inhibiting the Ang II axis.

In Chinese medicine, *Rheum rhabarbarum* is extensively used as an herbal drug [10]. One chief bioactive component from the rhubarb rhizome is rhein (4,5-dihydroxy-anthraquinone-2-carboxylic acid) [11, 12], which has diverse pharmacological functions such as inflammation resistance [13], tumor angiogenesis prevention [14], and hepatoprotection [15]. Its treatment potential has been investigated in animal experiments involving various other conditions, including chronic kidney disease [16], liver cancer [17], rheumatoid arthritis [18], and osteoarthritis [19]. Although the findings are encouraging, its specific mechanisms are not completely understood.

Oxidative stress contributes to CH and HF development [20]. Published work validated the dose–response repression of superoxide production by rhein [21]. Nemeikaite-Ceniene et al. demonstrated that rhein was a substrate for a wide variety of one electron-reducing flavoenzymes [22]. The oxidation resistance of rhein has been noted in the liver [17] and kidneys [16], vascular endothelial cells [23], and pancreatic cancer cells [24]. These studies imply that rhein can likely be used for protection against CH. Nevertheless, data backing this assumption are scarce. In the present study, we examined the assumption that rhein has a CH preventive role, which we accomplished through Ang II challenge in mice. We validated this by repressing the AMPK–FGF23 signaling pathway, where rhein inhibited Ang II-elicited pathological CH.

## Materials and methods

### Reagents and antibodies

Rhein (4,5-dihydroxyanthraquinone-2-carboxylic acid) is an anthraquinone compound isolated from rhubarb. Rhein [> 98% high-performance liquid chromatography (HPLC) purity] was purchased from Maclaurin (Shanghai, China). Antibodies against phosphor-AMPKα, AMPKαand GAPDH were purchased from Cell Signaling Technology (Boston, USA). The Anti-atrial natriuretic peptide (ANP), Anti-brain natriuretic peptide (BNP), Anti-FGF23, Anti-BAX and Anti-BCL-2 antibodies were from Abclonal (Wuhan, China).

### Animals and animal models

Approval was acquired for all animal experiments from The Quzhou Affiliated Hospital of Wenzhou Medical University. In every experiment, 8–10-week-old male mice weighing 24–28 g were placed in a 23 °C room on a 12-h light–dark cycle. For CH induction, the mice were infused subcutaneously daily for 4 weeks with 1.4 mg/kg Ang II (No. A9525; Sigma-Aldrich, St. Louis, MO, USA) by osmotic pumps (model 2004, ALZET) [25, 26]. Rhein was dissolved in 40% PEG 400–PBS solution by sonication. Regarding rhein therapy, 50 and 100 mg/kg rhein was injected for 4 weeks on consecutive days. The mice in the normal and Ang II-challenged groups received an equal volume of vehicle. 4 weeks post-Ang II infusion, the measurement of hypertrophy was performed according to previous research [27, 28]. The mice were killed by cervical dislocation, and the hearts and lungs were harvested and the heart weight normalized to tibia length (HW/TL), heart weight normalized to body weight (HW/BW) and lung weight normalized to eight body weight (LW/BW) were evaluated between vehicle- and rhein-treated mice.

### Isolation, treatment, and lentivirus transfection of primary cardiomyocytes (CMs) or cardiac fibroblasts (CFs)

Neonatal murine CMs and CFs were isolated from 2–3-day-old C57BL/6 mice. The initial step was cutting the cardiac tissue into approximately 1 mm^3^ pieces and their subsequent dissociation using collagenase II (0.07%) and trypsin (0.04%). This was followed by dish (100-mm) incubation of resuspended cells to allow 1-h adherence of noncardiac myocytes (primarily CFs) onto the plastic. Next, 5 × 10^5^ CMs were inoculated into each well of 6-well microplates for 48 h at 5% CO_2_ and 37 °C. After growing the cells in 10% FBS (fetal bovine serum) DMEM (Dulbecco’s modified Eagle’s medium) containing penicillin (100 U/mL)/streptomycin (100 μg/mL) for 48 h, the medium was replaced with serum-free medium for 12-h incubation before experimentation. Then, the cells were treated with low or high concentrations (5 and 25 μM, respectively) of rhein. Normal cells treated with dimethyl sulfoxide (DMSO) were used as the control. The cells were exposed for 24 h to varying concentrations of rhein and Ang II (1 μM) following a 2-h prior application of compound C (10 μM) to the medium.

### Immunofluorescence staining

The CM surface area was assessed with immunofluorescence staining with α-actinin (05-384, Merck Millipore). Briefly, CMs grown on coverslips underwent sequential 15-min fixation with 4% paraformaldehyde, 20-min permeabilization in 0.1% Triton X-100, 1-h (at shortest) blocking in 3% BSA (bovine serum albumin), and 2-h incubation with primary antibodies at ambient temperature, followed by washing and 1-h incubation with secondary antibodies under dark and ambient temperature conditions. After every step, the samples were washed three times with PBS. Lastly, the cells were rinsed three times and incubated with DAPI (C1005, Beyotime, Jiangsu, China). The cells were photographed with a confocal microscope (LSM510; Leica, Wetzlar, Germany) while the cell surface area was assessed with ImageJ 1.48v. The excitation wavelength of 405 nm (for exciting DAPI), 488 nm (for exciting the green fluorescein) and 568 nm (for exciting the red fluorescein) were used for imaging.

Primary CFs were fixed in 4% paraformaldehyde for 10 min and permeabilized with 0.1% Triton X-100 for 5 min to assess their phenotypic transformation. The CFs were blocked with 5% BSA at room temperature for 1 h and incubated with primary antibody against α-SMA at 4 °C overnight. The CFs were rinsed three times with PBS and incubated with the secondary antibody for 1 h at room temperature in the dark. The cells were visualized and photographed under a Nikon fluorescence microscope.

### Cell viability assay

CM and CF viability was assessed with Cell Counting Kit-8 (CCK-8, Beyotime). After 48-h culture, the CMs were treated for another 24 h with gradient concentrations (5, 10, 25, 50, 100 μM) of rhein in 96‐well plates. CCK‐8 (10 μL per well) was added to the plates and incubated for 2 h at 37 °C. The absorbance at 450 nm in each well was assessed with a microplate reader (Bio‐Rad).

### Wound healing assay and proliferation assay

For the wound healing assay, the CFs were seeded in 6-well plates containing serum-deprived medium for 24 h before wounding. When the CFs were at 90% confluence, a single scratch was made in the monolayer and the medium was replaced with fresh serum-deprived medium. Then, the migration speed was quantified.

CF proliferation was assayed using the CCK-8 assay kit. CFs (3 × 10^3^ cells) were seeded in serum-deprived medium for 24 h. After 24-h and 48-h culture, each well was incubated with CCK-8 solution. The absorbance was read using a spectrophotometer. The experiments involved five replicate wells per group.

### Detection of oxidative stress in vivo and in vitro

To detect reactive oxygen species (ROS) levels, the CMs or CFs and heart tissue were incubated for 45 min with dihydroethidium (DHE) dye (Sigma-Aldrich) at 37 °C in a light-protected humidified chamber after Ang II model establishment and rhein treatment. Superoxide anions in the cells were measured in a similar manner using DCFH-DA fluorescent probes (Beyotime). The results were observed with a fluorescence microscope (Carl Zeiss, Jena, Germany), followed by analysis with inverted fluorescent microscopy. Superoxide dismutase (SOD) and glutathione (GSH) activity in the CMs was assayed with the relevant biochemical kits (Beyotime).

### Echocardiographic measurements

At 4 weeks following Ang II treatment, the isoflurane (2%)-anaesthetized mice underwent echocardiography with a Vevo 2100 imaging platform (VisualSonics, Toronto, Canada). Parasternal short-axis images were captured at the mid-papillary muscle level to determine the LVEDd and LVESd (left ventricular end-diastolic and end-systolic diameters). The LV ejection fraction (EF%), considered a systolic function indicator, was determined as follows: (LVEDV − LDESV)/LVEDV × 100.

### Histological analysis

The mouse heart tissue underwent sequential 24-h paraformaldehyde (4%) fixation, paraffin-embedding, and sectioning into 5-μm pieces. Then, the sections were stained with hematoxylin and eosin (H&E, Servicebio, Wuhan, China) or picrosirius red (PSR) (Servicebio) according to standard procedures for histopathological purposes and analysis of collagen deposition, respectively. The myocyte cross-sectional areas and fibrotic areas were determined with Image-Pro Plus digital image analyzing software (ver. 6.0).

### Quantitative real-time PCR (RT-qPCR)

Total RNA was extracted from the CMs, CFs, or cardiac tissue using TRIzol (TaKaRa, Shiga, Japan) as per the manufacturer’s protocol. The total mRNA (1 μg) was reverse-transcribed into complementary DNA using the Maxima H Minus First Strand cDNA Synthesis Kit (Thermo Fisher Scientific). The qPCR was performed in accordance with standard procedures and the transcript levels were standardized to *GAPDH*.

### Western blotting

Proteins from the cardiac tissue or cultured cells were sampled with radioimmunoprecipitation assay buffer (Beyotime). Equivalent quantities (40–60 μg) of protein were separated by sodium dodecyl sulfate–polyacrylamide gel electrophoresis (7.5–12.5%), and then transferred to polyvinylidene fluoride membranes. The membranes were blocked for 1 h with 5% skimmed milk in TBST at ambient temperature and were incubated overnight with the relevant primary antibodies. Subsequently, the blots were incubated for 1 h with horseradish peroxidase (HRP)-conjugated secondary antibodies (1:1000 dilution) at ambient temperature. The protein levels were quantified with the Quantity One software package (Bio-Rad).

### Data and statistical analysis

The results were all presented as the means ± SDs. A normal distribution test was performed to determine whether a parametric or non-parametric test was conducted. Student’s t-test was used for comparisons between two groups. Comparisons of more than 3 groups were carried out by one-way ANOVA. All statistical analyses were performed using GraphPad Prism 8.0 software. Differences were regarded as significant when P < 0.05.

## Results

### Rhein inhibited Ang II-induced CH and improved cardiac function in mice

**Fig. 1:**
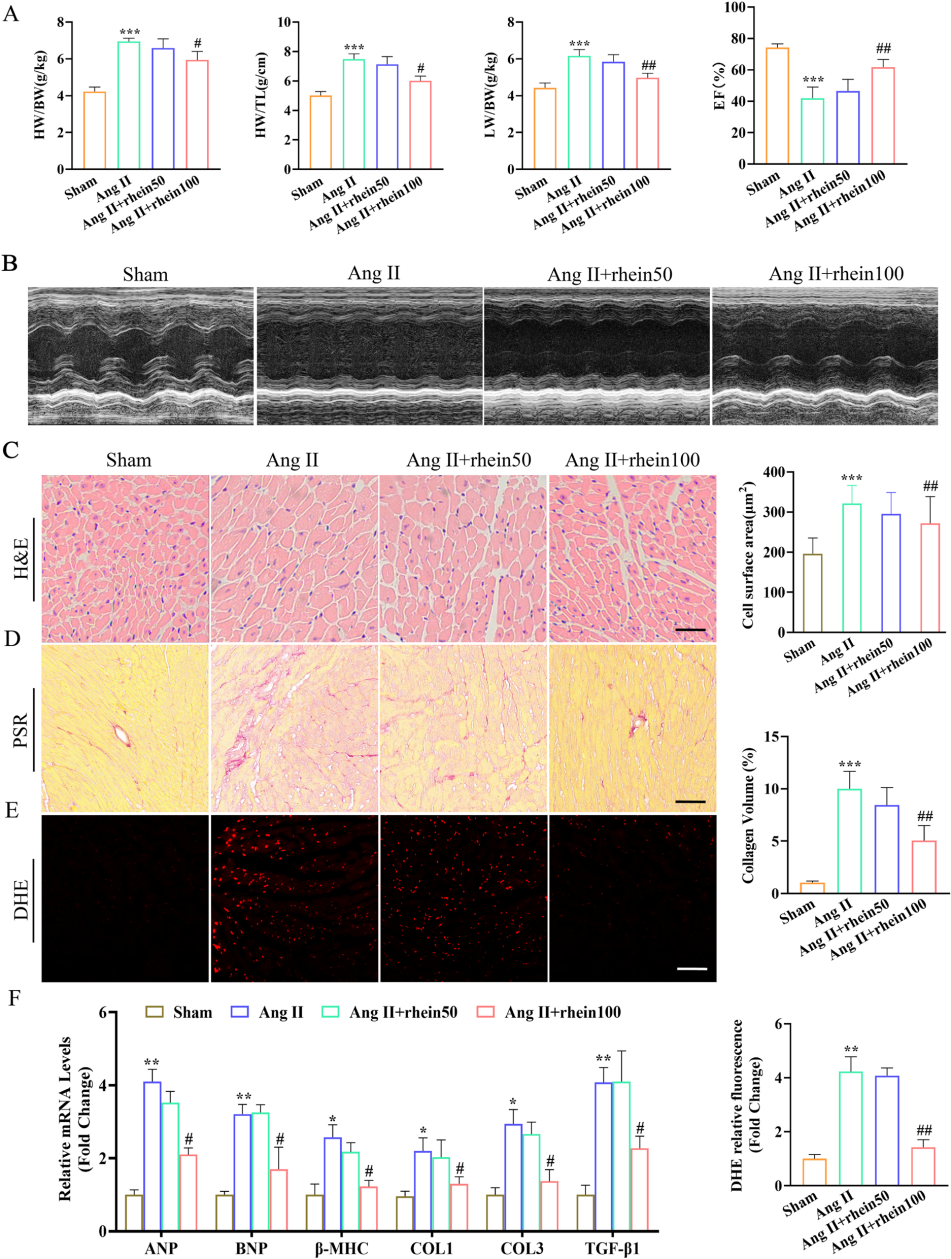
Rhein suppressed Ang II-induced cardiac remodeling in mice. **A** The heart mass/body mass, heart mass/TL, and LW/BW ratios (n = 7 per group). **B** Cardiac function was assessed by the EF% (scale bar = 200 μm; n = 7 per group). **C** Representative images of H&E-stained LV transverse sections (scale bar = 50 μm; n = 6 per group). **D** PSR-stained LV cross-sections (scale bar = 50 μm; n = 5 per group). **E** Representative micrographs and quantification of DHE-stained superoxide anion levels (n = 5 per group). **F** ANP, BNP, β-MHC, TGF-β_1_, collagen I, and collagen III mRNA levels in mouse hearts (n = 5 per group). *P < 0.05, **P < 0.01, and ***P < 0.001 vs. the sham group; ^#^P < 0.05 and ^##^P < 0.01 vs. the Ang II group

To examine the in vivo effect of rhein on CH and contractile functionality, wild-type mice were given either vehicle or rhein (50 or 100 mg/kg) daily and were infused with sham or chronic Ang II subcutaneously for 4 weeks. Rhein reversed Ang II-induced CH in an obvious manner (Fig. 1A), which manifested as decreased heart weight normalized to tibia length (HW/TL), HW normalized to body weight (HW/BW) and lung weight normalized to body weight (LW/BW). The role of rhein in cardiac functionality was examined by echocardiography of the Ang II-induced mice. Higher-concentration rhein protected against the Ang II-induced CH prominently in contrast to that in the Ang II group, as manifested by elevated LVEF (Fig. 1B). H&E staining revealed that the Ang II-induced mice exhibited greater cross-sectional areas of CMs and LV wall thickness than the sham group, while rhein prominently weakened these alterations (Fig. 1C). Cardiac tissue compliance can be weakened drastically by extracellular matrix (ECM) (collagen and fibronectin) synthesis, which can also cause contractile function disorder [29]. The role of rhein in collagen synthesis was assessed by immunohistology and RT-qPCR. PSR staining revealed that the rhein-treated mice had less collagen deposition than Ang II-induced mice (Fig. 1D). The pivotal role of oxidative stress in CH and HF is universally recognized [20]. Rhein prominently repressed the elevated cardiac tissue expression of total ROS triggered by Ang II (Fig. 1E). Consistent with this, the Ang II group demonstrated elevated levels of hypertrophic markers [ANP (atrial natriuretic peptide), BNP (brain natriuretic peptide), and β-MHC] and fibrotic markers (TGF-β_1_, collagen I, and collagen III), while their expression was inhibited in the rhein-treated mice (Fig. 1F). Taken together, the findings validated the protective effect of rhein against Ang II-induced CH.

### Rhein suppressed Ang II-induced CH and oxidative stress in vitro

**Fig. 2:**
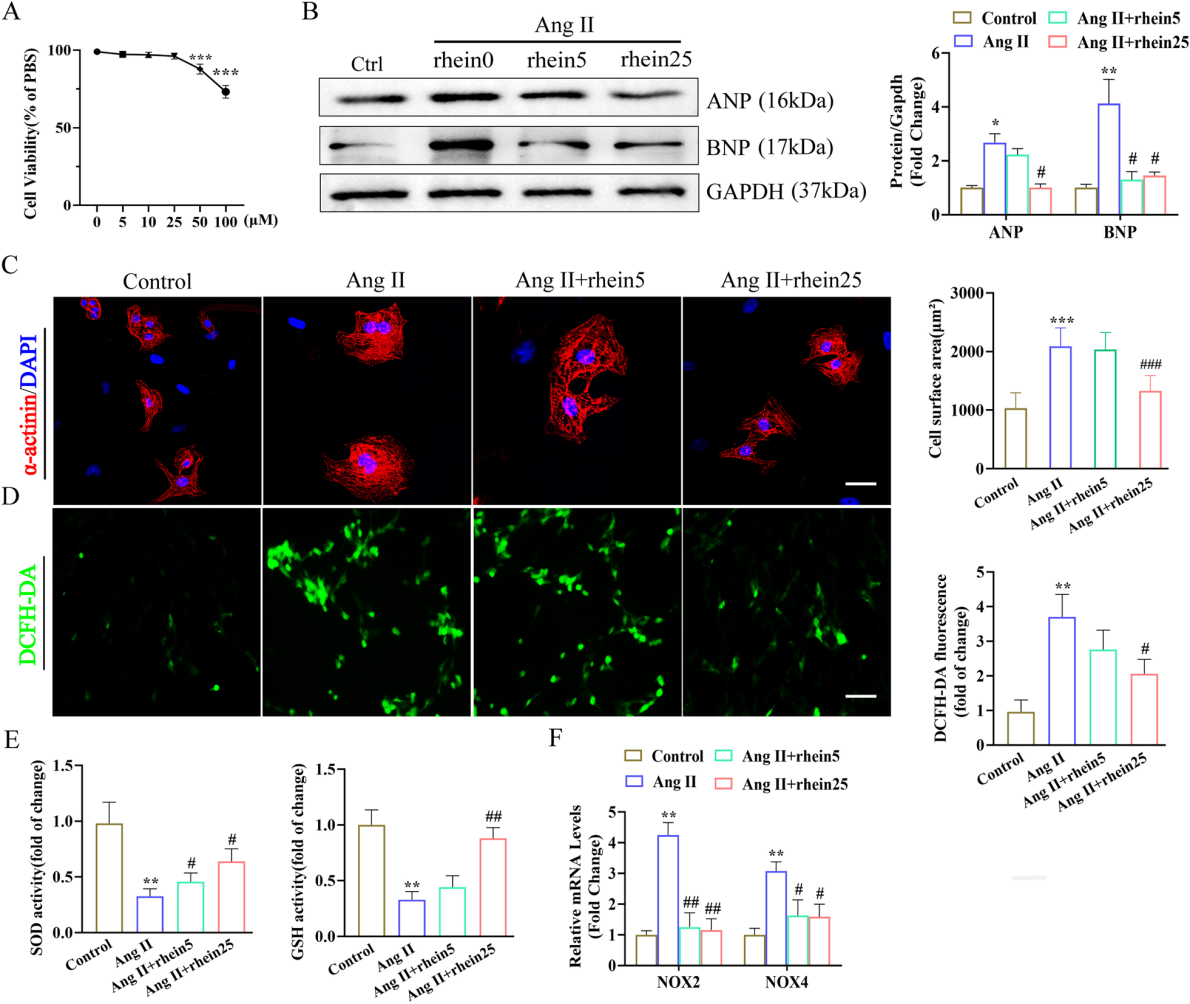
Rhein reduced Ang II-induced CM hypertrophy and oxidative stress. **A** The 24-h exposure of CMs to rhein (0, 5, 10, 25, 50, and 100 μM). Cellular viability was examined by CCK-8 assay (n = 5 per experiment). **B** Immunoblots and quantification of ANP and BNP expression in CMs induced with Ang II and treated with rhein for 24 h. **C** Representative micrographs of CMs stained with α-actinin (red) and DAPI (blue) (scale bars = 20 μm; n ≥ 50 cells per group). **D** DCFH-DA detection of ROS (n = 5 per group). **E**, SOD and GSH activity (n = 5 per group). **F**, RT-qPCR quantification of NOX2 and NOX4 mRNA expression levels (n = 5 per experiment). *P < 0.05 and **P < 0.01 vs. the control group; ^#^P < 0.05 and ^##^P < 0.01 vs. the Ang II group

We investigated whether rhein affects CM hypertrophic responses. At the 0, 5, 10, and 25 μM, rhein did not affect CM viability (Fig. 2A). Based on the 24-h timepoint of a cell viability assay, 5 and 25 μM rhein were chosen to examine its role in CMs. Western blotting revealed that Ang II elevated CM expression of *BNP* and *ANP*, while rhein pre-treatment repressed the Ang II-induced expression of these genes (Fig. 2B). α-Actinin staining of the CMs revealed that higher concentrations of rhein (25 μM) caused a significant decrease in CM size (Fig. 2C). The indispensable function of rhein in oxidative stress modulation has been demonstrated previously [16, 17, 23]. DCFH-DA staining revealed that rhein blocked Ang II-induced ROS production in the CMs (Fig. 2D). Furthermore, rhein treatment restored antioxidase activity, including that of SOD and GSH, in the Ang II-treated CMs (Fig. 2E). Consistent with this, NOX2 and NOX4 levels increased after Ang II stimulation in the CMs, while rhein reversed these changes in an obvious manner (Fig. 2F). In addition, western blot analysis showed that rhein inhibited the Ang II-induced increase in BAX expression and increased the expression of the antiapoptotic molecule BCL-2 in CMs (Additional file 1: Fig. S1). Collectively, rhein prevented the CM hypertrophy elicited by Ang II in vitro.

### Rhein attenuated Ang II-Induced CF activation in vitro

**Fig. 3:**
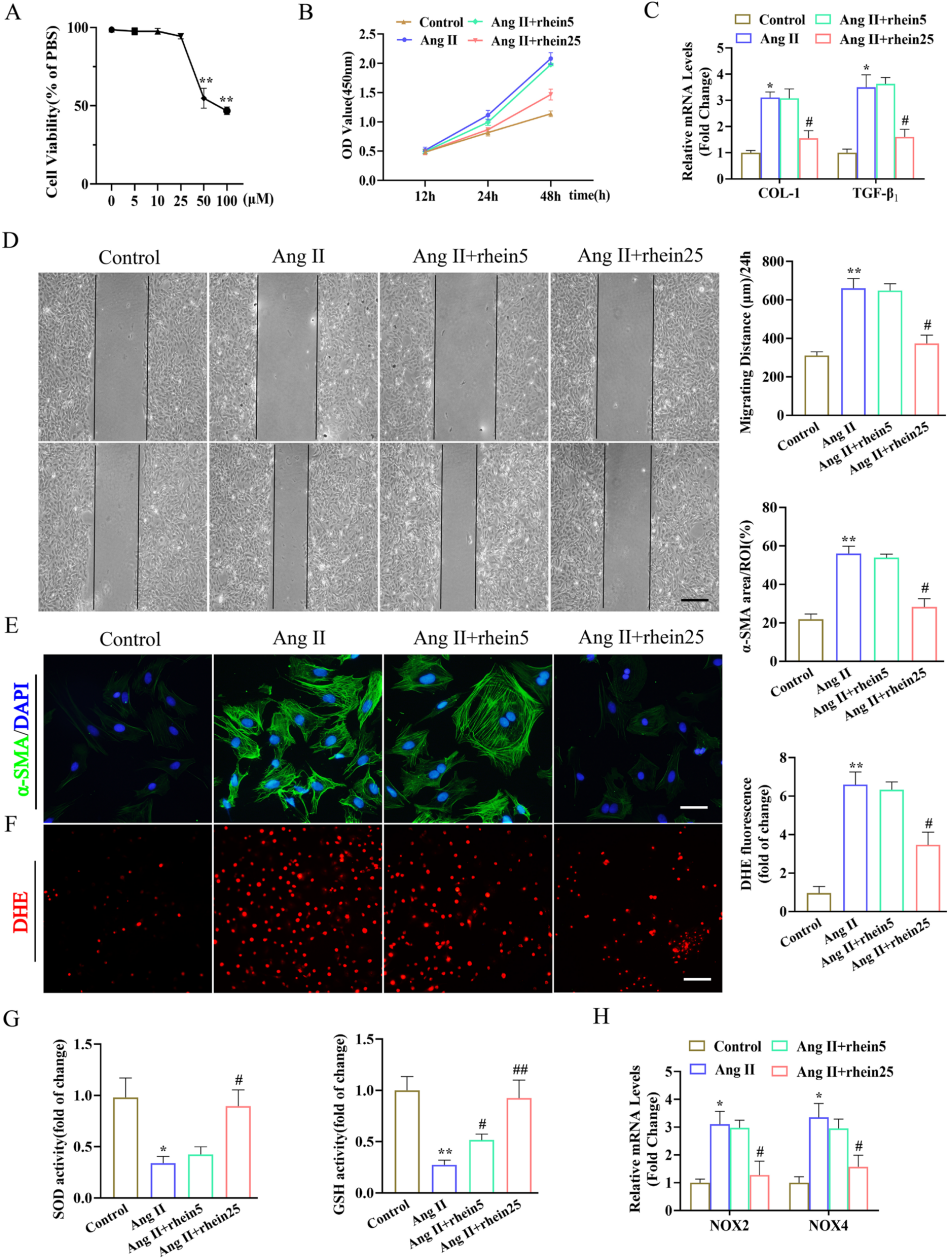
Rhein attenuated Ang II-induced CF activation in vitro. **A** The 24-h exposure of CFs to rhein (0, 5, 10, 25, 50, and 100 μM). Cellular viability was examined by CCK-8 assays (n = 5 per experiment). **B** CCK-8 assay quantification of CF proliferation (n = 5 per experiment). **C** RT-qPCR quantification of collagen I and TGF-β_1_ mRNA levels (n = 5 per experiment). **D** Wound repair assays for assessing CF migration ability (n = 5 per experiment). **E** Immunofluorescence staining of α-SMA expression (green) and the nuclei (DAPI, blue) (n = 5 per group, ≥ 20 fields per group; scale bar = 100 μm). **F**, Immunofluorescence DHE staining of superoxide anion levels (n = 5 per group). **G**, SOD and GSH activity (n = 5 per group). **H**, RT-qPCR quantification of NOX2 and NOX4 mRNA levels (n = 5 per experiment). *P < 0.05 and **P < 0.01 vs. the control group; ^#^P < 0.05 and ^##^P < 0.01 vs. the Ang II group

CFs were cultured and treated with rhein to investigate its role in cardiac fibrosis. Based on a cell viability assay, we selected 5 and 25 μM rhein to examine its role in CFs (Fig. 3A). Rhein suppressed CF conversion to myofibroblasts, as indicated by inhibition of the CF proliferative and migration capabilities (Fig. 3B, D) and decreased α-SMA, collagen I, and TGF-β_1_ expression (Fig. 3C, E). We investigated whether rhein could also inhibit ROS production in the CFs. Rhein dose-dependently suppressed ROS levels and promoted SOD and GSH activity (Fig. 3F, G). Consistent with this, rhein treatment markedly inhibited the Ang II-induced upregulation of NOX2 and NOX4 expression (Fig. 3H). We also found that rhein suppressed the Ang II-induced increase in BAX expression and enhanced the expression of the antiapoptotic molecule BCL-2 in CFs by western blot analysis (Additional file 1: Fig. S1). Therefore, these data indicated that rhein significantly suppressed Ang II-induced CF activation and oxidative stress.

### RNA sequencing emphasized the biological processes and target molecules regulated to attenuate cardiac remodeling by rhein

**Fig. 4:**
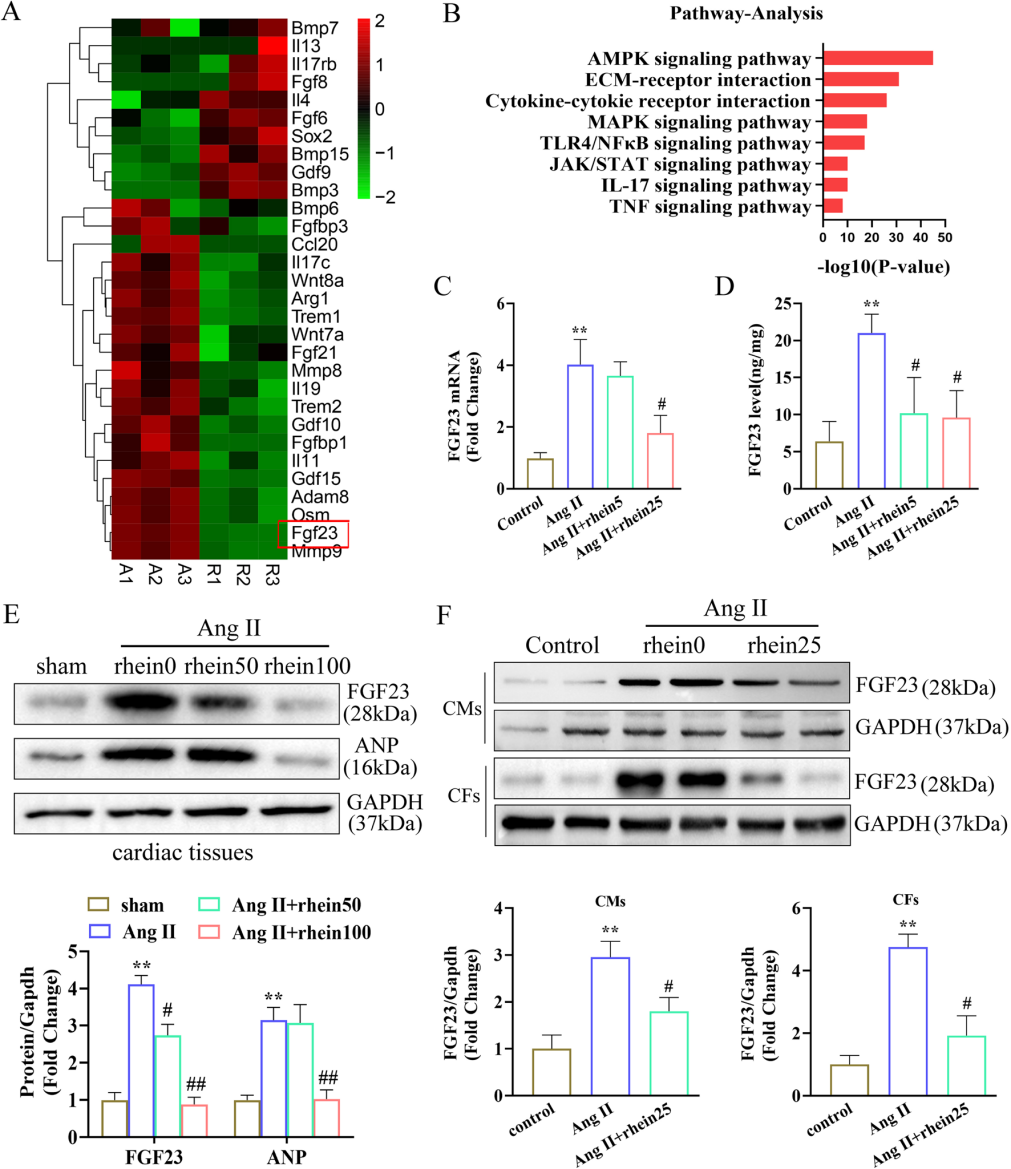
RNA sequencing emphasized the biological processes and target molecules regulated to attenuate cardiac remodeling by rhein. **A** Heat map showing the expression patterns of differentially expressed genes. **B** KEGG analysis. **C**, **D** RNA sequencing results were validated by qPCR (n = 5 per group) and enzyme-linked immunosorbent assay (n = 5 per group). **E** Western blot analysis and quantification of FGF23 expression in cardiac tissue (n = 4 per group). **F** Western blot analysis and quantification of FGF23 expression in CMs or CFs (n = 4 per group). **C**, **D** **P < 0.01 vs. the control group; ^#^P < 0.05 vs. the Ang II group. **E** **P < 0.01 vs. the sham group; ^#^P < 0.05 and ^##^P < 0.01 vs. the Ang II group

We performed genome-wide transcriptional profiling to investigate the potential mechanisms of rhein on cardiac remodeling. For RNA sequencing, fold changes ≥ 1.5 and P ≤ 0.05 were considered statistically significant. A total of 578 genes were down regulated significantly with the applications of Rhein in CH models (Fig. 4A). By KEGG analysis, the genes were clustered to be related with the AMPK, TLR4–NF-κB, and MAPK signaling pathways (Fig. 4B), indicating the CH derived pathological changes could be alleviated by using Rhein. We performed functional clustering analysis of these differentially expressed genes. Gene Ontology (GO) enrichment analysis revealed that these target genes were related to energy metabolism, positive regulation of collagen biosynthetic processes, and the oxidative stress response. To investigate the mechanism by which rhein inhibited cardiac remodeling, we focused on the biological processes enriched among the 578 genes and observed significant differences in *FGF23* expression. Previous studies have shown that *FGF23* is closely related to the above biological functions and significantly promotes cardiac hypertrophy and fibrosis [30], idiopathic pulmonary fibrosis [31], and renal fibrosis [32]. We verified the altered expression of *FGF23* in vivo and in vitro (Fig. 3C–F). Rhein inhibited *FGF23* expression in the CFs, CMs, and heart tissue. Therefore, we think that the protective effects of rhein on cardiac remodeling were associated with the inhibition of *FGF23* expression.

### FGF23 overexpression weakened the protective effects of rhein in CFs and CMs

**Fig. 5:**
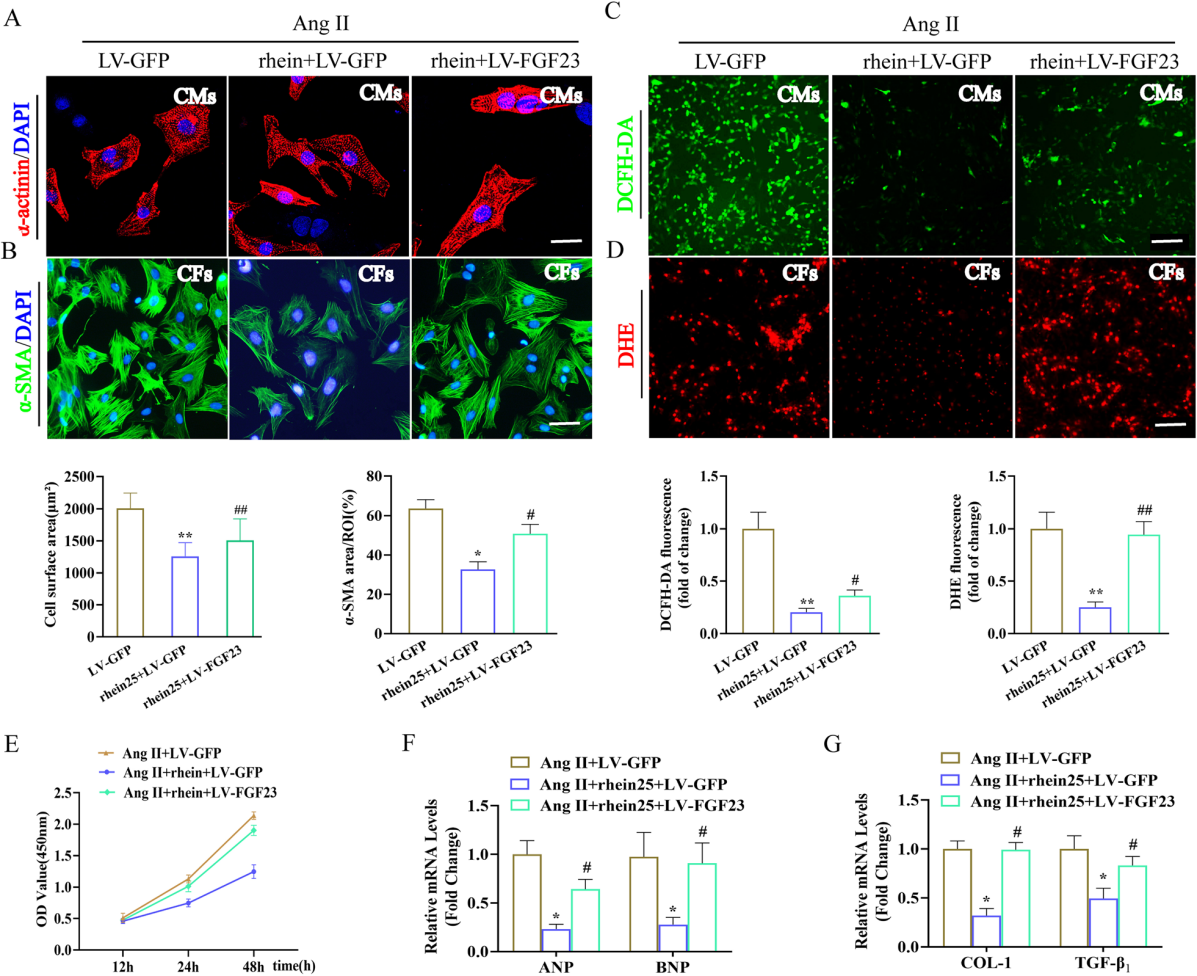
*FGF23* overexpression obviously weakened the protective effects of rhein in vitro. **A** Representative micrographs of CMs stained with α-actinin (red) and DAPI (blue) (scale bars = 20 μm; n ≥ 50 cells per group). **B** Immunofluorescence staining of α-SMA expression (green) and nuclei (DAPI, blue) (n = 5 per group, ≥ 20 fields per group; scale bar = 100 μm). **C** Immunofluorescence DCFH-DA staining of ROS levels in CMs (n = 5 per group). **D** Immunofluorescence DHE staining of ROS levels in CFs (n = 5 per group). **E** CCK-8 quantification of CF proliferation (n = 5 per experiment). **F, G**, RT-qPCR quantification of ANP, BNP, collagen I, and TGF-β_1_ mRNA levels (n = 5 per experiment). *P < 0.05 and **P < 0.01 vs. the Ang II + LV-GFP group; ^#^P < 0.05 and ^##^P < 0.01 vs, the Ang II + rhein25 + LV-GFP group

We explored whether FGF23 could reverse the protective effects of rhein in vitro. CFs and CMs were infected with *FGF23*-overexpressing lentiviruses (LV-FGF23) to assess its role in cardiac remodeling (Additional file 1: Fig. S2A, B). *FGF23* overexpression significantly weakened the protective effects of rhein compared with the rhein treatment group, indicated by blocking the inhibitory effects of rhein on Ang II-induced CM hypertrophy (Fig. 5A) and CF proliferation (Fig. 5E) and phenotypic transformation (Fig. 5B). Furthermore, *FGF23* overexpression inhibited the protection of rhein against Ang II-induced ROS generation in the CFs and CMs (Fig. 5C, D). *FGF23* overexpression suppressed the rhein-mediated negative regulation of the mRNA levels of ANP and BNP in the CMs and collagen I and TGF-β_1_ in the CFs (Fig. 5F, G). These results suggested that *FGF23* overexpression markedly abolished the protection of rhein in CFs and CMs.

### *FGF23* overexpression attenuated the rhein-mediated inhibitory effects on cardiac remodeling in mice

**Fig. 6:**
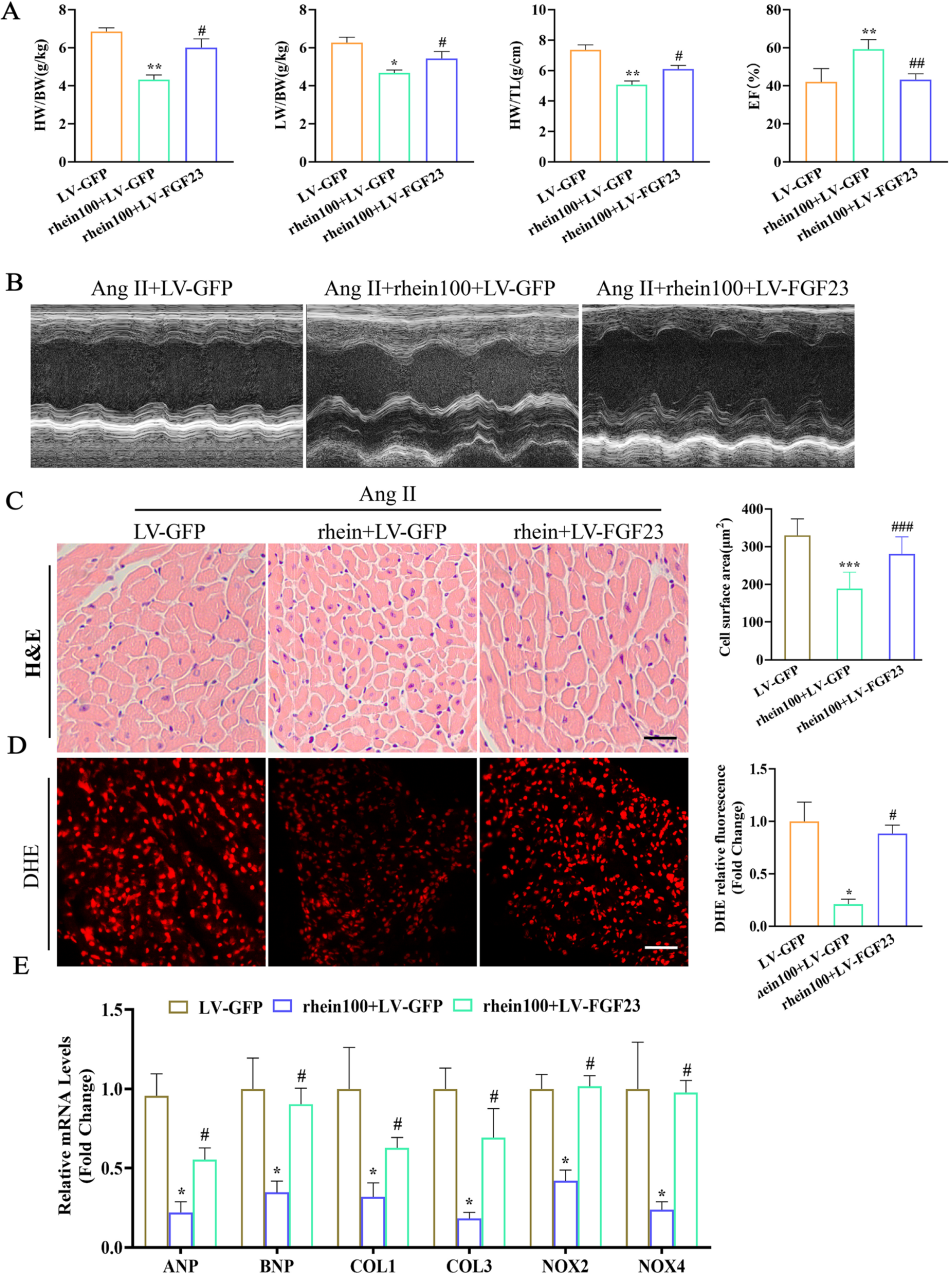
*FGF23* overexpression attenuated the rhein-mediated inhibitory effect on cardiac remodeling in mice. **A** The heart mass/body mass, heart mass/TL, and LW/BW ratios (n = 6 per group). **B** Cardiac function was assessed by the EF% (scale bar = 200 μm; n = 6 per group). **C** Representative images of H&E-stained LV transverse sections (scale bar, 50 μm; n = 6 per group). **D** Representative micrographs and quantification of the DHE-stained superoxide anion levels (n = 5 per group). **E** ANP, BNP, collagen I, collagen III, NOX2, and NOX4 mRNA levels in mouse hearts (n = 5 per group). *P < 0.05, **P < 0.01, and ***P < 0.001 vs. the Ang II + LV-GFP group; ^#^P < 0.05, ^##^P < 0.01, and ^***^P < 0.001 vs. the Ang II + rhein100 + LV-GFP group

To assess whether *FGF23* is involved in Ang II-induced cardiac remodeling and dysfunction after rhein treatment, *FGF23* was overexpressed in cardiac tissue by injecting LV-FGF23 (Additional file 1: Fig. S2C). Rhein treatment obviously improved Ang II-induced cardiac contractile dysfunction as indicated by the decreased HW/TL, HW/BW, and LW/BW ratios (Fig. 6A); increased EF% (Fig. 6B); attenuated CM hypertrophy (Fig. 6C); ROS levels (Fig. 6D); and the mRNA expression of ANP, BNP, collagen I, and collagen III (Fig. 6E), whereas *FGF23* overexpression weakened the protective effects of rhein. Taken together, these results suggested that rhein blocked cardiac remodeling by suppressing *FGF23* expression.

### Rhein activated AMPK activity and expression leading to blocked *FGF23* expression

**Fig. 7:**
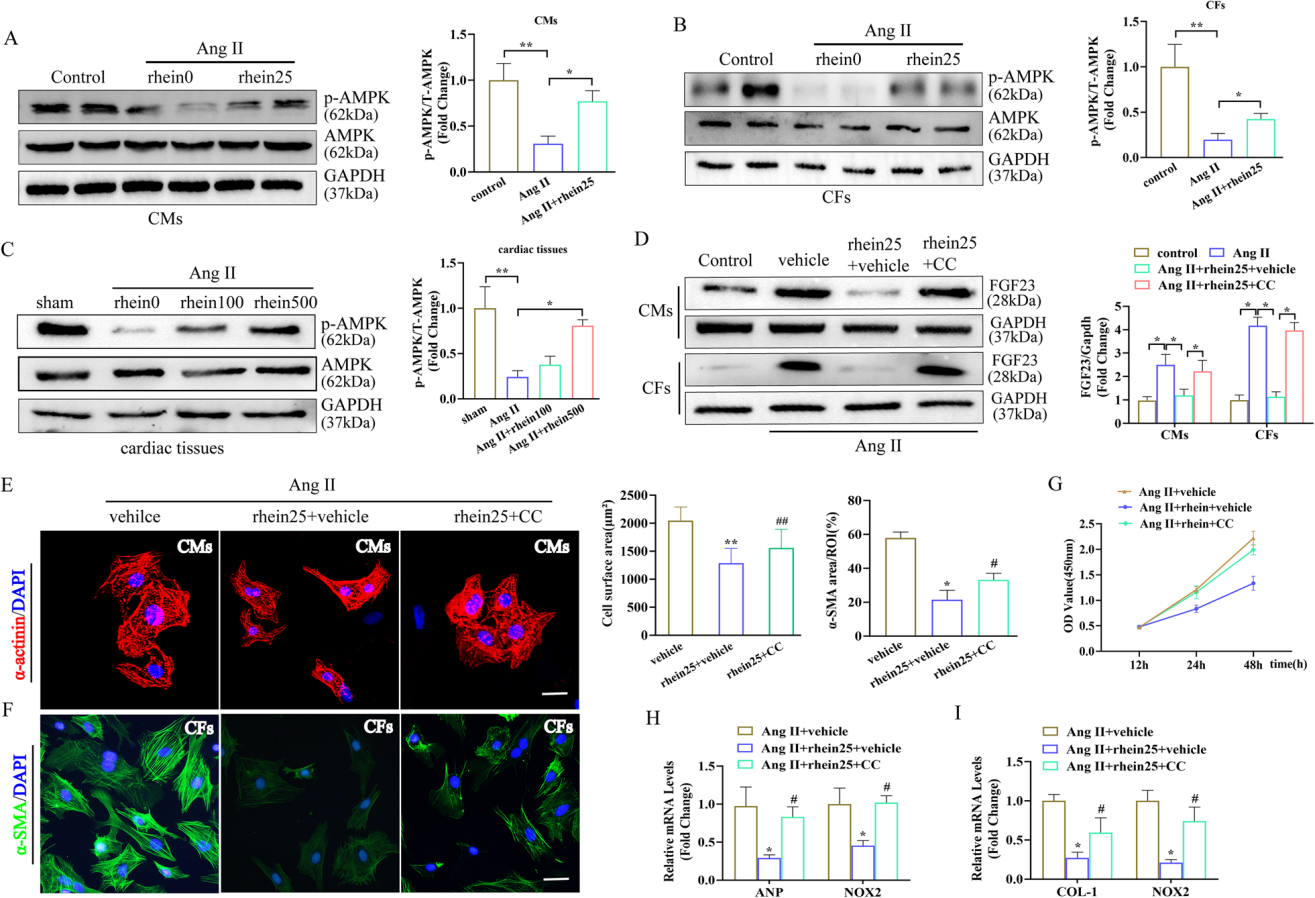
Rhein activated AMPK activity and expression leading to blocked *FGF23* expression. **A**–**C** Representative immunoblots and quantitative results for the overall and p-AMPK expression (n = 4 per group). **D** Representative immunoblots and quantitative results for FGF23 expression (n = 4 per group). **E** Representative micrographs of CMs stained with α-actinin (red) and DAPI (blue) (scale bars = 20 μm; n ≥ 50 cells per group). **F** Immunofluorescence staining of α-SMA expression (green) and nuclei (DAPI, blue) (n = 5 per group, ≥ 20 fields per group; scale bar = 100 μm). **G** CCK-8 assay quantification of CF proliferation (n = 5 per experiment). **H**, **I** RT-qPCR quantification of ANP, collagen I, and NOX2 mRNA levels (n = 5 per experiment). **A**–**D** *P < 0.05 and **P < 0.01; E-I, *P < 0.05 and **P < 0.01 vs. the Ang II + vehicle group; ^#^P < 0.05 and ^##^P < 0.01 vs. the Ang II + rhein25 + vehicle group

AMPK is a regulator of *FGF23* production and the inhibition of AMPK activity promoted *FGF23* transcription [33]. Therefore, we tested the AMPK expression levels to illustrate whether the protective effect of rhein on cardiac remodeling was involved in AMPK activity and expression in vitro. We pretreated CMs and CFs with the AMPK-specific inhibitor compound C and then stimulated them with Ang II. Ang II stimulation inhibited AMPK activation but this effect was reversed by rhein (Fig. 7A–C). Compound C abolished the effects of rhein-induced reduction of *FGF23* expression in the CMs and CFs (Fig. 7D). Therefore, we concluded that rhein inhibited Ang II-induced *FGF23* transcription by activating AMPK activity. Moreover, compound C weakened the protective effects of rhein against Ang II-induced CM hypertrophy (Fig. 7E) and CF proliferation and phenotypic transformation (Fig. 7F, G). Similar results were observed for the ANP, collagen I, and NOX2 expression levels in the treatment groups (Fig. 7H, I). In summary, these findings suggested that rhein suppressed Ang II-induced cardiac remodeling by inhibiting AMPK–FGF23 signaling.

**Fig. 8:**
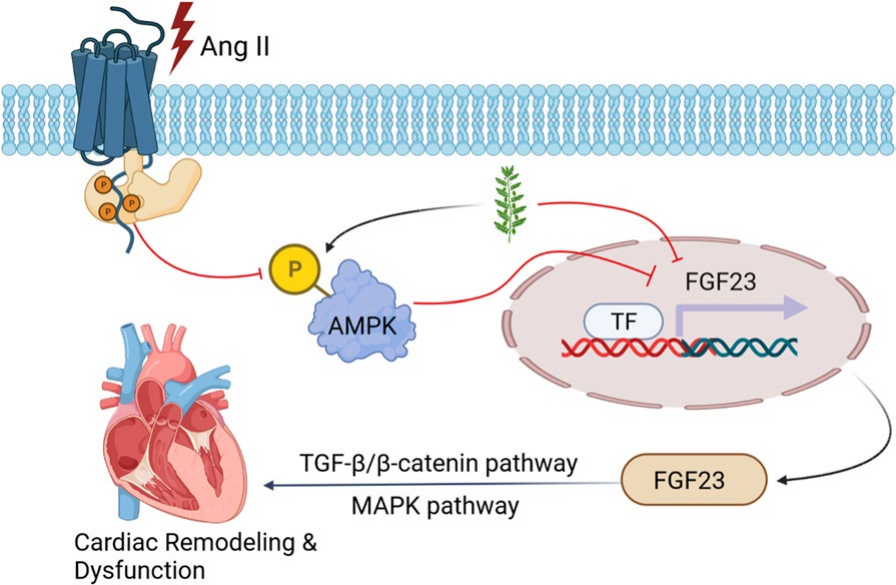
Schematic model of rhein-mediated cardioprotection in Ang II-induced cardiac remodeling by inhibiting AMPK–FGF23 signaling

Model illustrating that rhein-mediated cardioprotection in Ang II-induced cardiac hypertrophy by Modulating AMPK–FGF23 Signaling (Fig. 8).

## Discussion

The present research explored the role of rhein in Ang II-elicited CH in vitro and myocardial hypertrophy in vivo. Rhein supplementation inhibited Ang II-induced CM hypertrophy, CF phenotypic transformation, and cellular ROS production. In vivo, rhein supplementation significantly suppressed Ang II-induced CH, oxidative stress, and cardiac fibrosis in mice and ameliorated their cardiac functionality. Mechanistic experimentation revealed that rhein suppressed *FGF23* expression in Ang II-induced cardiac remodeling in an obvious manner. Furthermore, *FGF23* overexpression inhibited the protective effects of rhein in pathological cardiac remodeling. Importantly, rhein could reduce FGF23 expression, mainly through the activation of AMPK. In summary, rhein can serve as a potential therapy of CH via the AMPK–FGF23 axis.

Experimental and clinical works in the last few decades have yielded extensive evidence that ROS can affect many kinds of pivotal cardiac maladaptation traits, such as the hypertrophic reaction, ECM remodeling, and contractile dysfunction [20, 34]. ROS is derived from many sources in the heart, such as cyclooxygenase, NADPH, lipoxygenase, mitochondria, and xanthine oxidase [35]. In the course of CH pathology, NADPH cytoplasmic modulatory subunits are transferred to the plasma membrane, thereby stimulating oxidase [36]. Pathological CH is linked to ROS surplus, which eventually leads to CM apoptosis and heart failure [26]. Although oxidative stress exerts a pivotal effect on CH occurrence and progression in a wide range of preclinical models, there is no direct targeted therapy for ROS into the clinical field. In our study on the rhein-mediated protective mechanism against pathological CH, we demonstrated the prominent suppressive role of rhein in oxidative stress following hypertrophic stress. Liu et al. demonstrated that rhein increased H9C2 cell viability and weakened the ROS production and apoptosis of cells under hypoxia/reoxygenation injury exposure [37]. Heo et al. reported that rhein inhibited monocyte migration through decreased ROS generation and initiation of NADPH oxidase p47 (phox) [38]. In addition, rhein is believed to facilitate diabetic nephropathy involved with ameliorating oxidative stress [39]. Here, rhein treatment restored antioxidase activity, including that of SOD and GSH, in Ang II-treated CMs and CFs. The elevated NOX2 and NOX4 protein levels after Ang II stimulation in the CMs and CFs were obviously reversed by rhein. Consistent with these findings, rhein significantly suppressed neuronal oxidative stress in Alzheimer disease [40], hydrogen peroxide-induced oxidative stress in intestinal epithelial cells [41], and ATP-triggered inflammatory responses in rheumatoid rat fibroblast-like synoviocytes [18]. Further research is needed to examine whether rhein has similar effects on ROS derived from the mitochondria or other sources.

Released primarily by osteocytes, FGF23 is a hormone that regulates phosphate and vitamin D metabolism [42]. FGF23 plays a prominent role in regulating CH and HF [26]. Circulating FGF23 was associated with CH in patients with chronic kidney and heart disease [43]. In vitro, FGF23 obviously enhanced CM size and hypertrophic gene marker expression [44]. Recent studies have suggested that FGF23 is closely related to oxidative stress. Excessive FGF23 induced endothelial dysfunction mainly by promoting oxidative stress [45]. Dong et al. reported that FGF23 induced atrial fibrosis by enhancing ROS levels [46]. However, the mechanism of FGF23 in cardiac remodeling remains unclear. We found increased FGF23 levels in the CMs, CFs, and heart tissue in response to Ang II stimulation, while the effects were reversed by rhein. Furthermore, FGF23 overexpression weakened the protective effects of rhein in mice following Ang II exposure. We also found that *FGF23* overexpression suppressed the rhein-mediated reduction of ROS generation in CFs and CMs in an obvious manner.

f some activating signaling pathways has been reported, such as that of AMPK–mTOR, MAPK, and NF-κB. In this work, rhein promoted phosphorylated (p)-AMPK levels prominently. Capable of sensing cellular energy and nutrient status, AMPK directly mediates multiple metabolic processes, such as fatty acid oxidation and glycolysis [47]. AMPK can also mediate other metabolic processes, including that of PI3K, mTOR, and SIRT1 [48]. AMPK deficiency aggravates Ang II-elicited CH, myocardial infarction, and pressure overload [49]. Repression of AMPK activity in CH through mTOR signaling has been demonstrated previously [50]. In murine renal tubular cells, rhein inhibited autophagy by critical molecule regulation in the AMPK-reliant mTOR pathways [51]. Rhein improved pemetrexed efficacy in managing non–small cell lung cancer by modulating the PI3K–AKT–mTOR pathway [52]. Many studies have demonstrated the initiation of AMPK by oxidative stress, implying an extra redox-sensing action of AMPK. A769662, the AMPKα2 activator, exerts a cardioprotective role against ROS production and apoptosis [53]. Ischemia/reperfusion injury can be resisted by metformin-mediated activation through eNOS phosphorylation at Ser1177, and PGC-1α stimulation [54]. Consistent with the above findings, we proved that rhein suppressed CH by inhibiting AMPK–mTOR signaling. A selective AMPK inhibitor, compound C overturned the declined p-mTOR expression mediated by rhein and the relief of Ang II-elicited CM hypertrophy by rhein. Consistent with this, it impaired the protective effect of rhein on oxidative stress in vitro. Therefore, rhein is a potential medication for targeting AMPK to treat cardiovascular diseases and prevent HF.

Several limitations of our study should be considered to clarify the causative mechanisms of the protective actions of rhein. First, our research was implemented at a mere two doses. The histological concentration, distribution, and pharmacodynamics of rhein are unclear. Further studies are required to identify the optimal dosage and administration method for rhein. Second, other signaling pathways may be associated with CH, such as the PI3K–AKT, TGF-β_1_–SMAD, and JAK–STAT pathways. Further investigation is required to examine whether rhein can inhibit CH through the abovementioned signaling pathways. Third, previous studies indicated multiple pharmacological functions of rhein, such as inflammatory resistance, anti-apoptotic, and autophagy promotion. Further studies are needed to exclude the probable systemic effects contributing to the hypertrophic resistance.

Conclusively, this work presented the first evidence that rhein prominently attenuated the pathological processes of cardiac remodeling (e.g., oxidative stress, hypertrophy, cardiac dysfunction, fibrosis) after Ang II exposure. Mechanistically, rhein supplementation prevented hypertensive cardiac remodeling by inhibiting AMPK–FGF23 signaling. Therefore, rhein be a potential preventive drug for hypertensive cardiac remodeling.

## Supplementary Information

**Additional file 1: Figure S1.** The evaluation of pro/anti-apoptotic markers referred to in vitro analysis. A–B, Immunoblots and quantification of BAX and BCL-2 expression in CMs and CFs. *P < 0.05.

**Additional file 2: Figure S2.** Verification of *FGF23* overexpression. A–C, Immunoblots and quantification of *FGF23* expression in CMs, CFs, and cardiac tissue.*P < 0.05 and **P < 0.01.

## Data Availability

All data generated in this study will be provided by the corresponding author upon request.
