# Supplementary figures and images for: Rhein attenuates angiotensin II-induced cardiac remodeling by modulating AMPK–FGF23 signaling
Source: J Transl Med. 2022 Jul 6;20:305. doi: 10.1186/s12967-022-03482-9 (PMC9258170; doi:10.1186/s12967-022-03482-9)

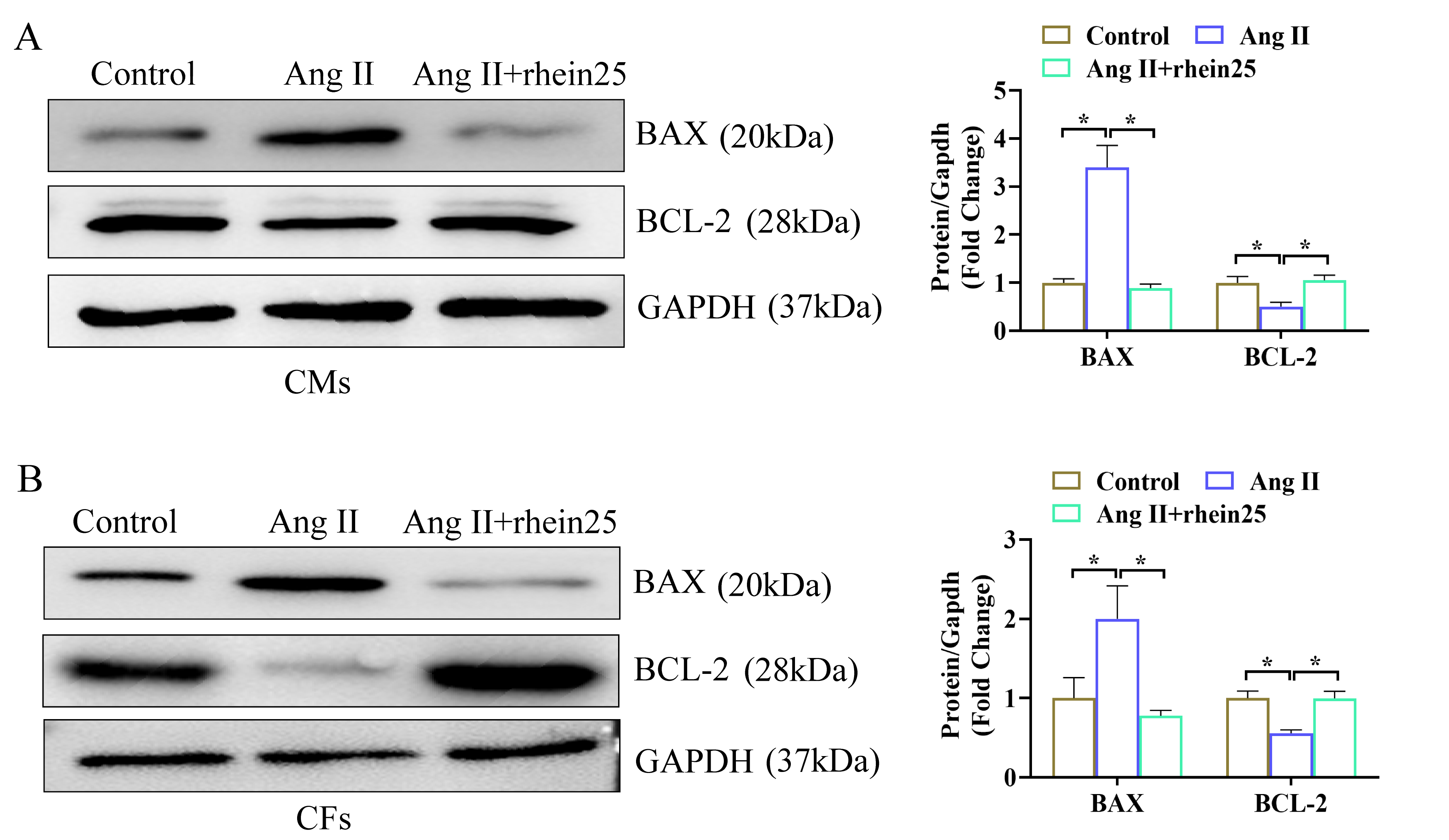

Supplement: Supplementary file 1 — Additional file 1: Figure S1. The evaluation of pro/anti-apoptotic markers referred to in vitro analysis. A–B, Immunoblots and quantification of BAX and BCL-2 expression in CMs and CFs. *P < 0.05. [file 12967_2022_3482_MOESM1_ESM.jpg]

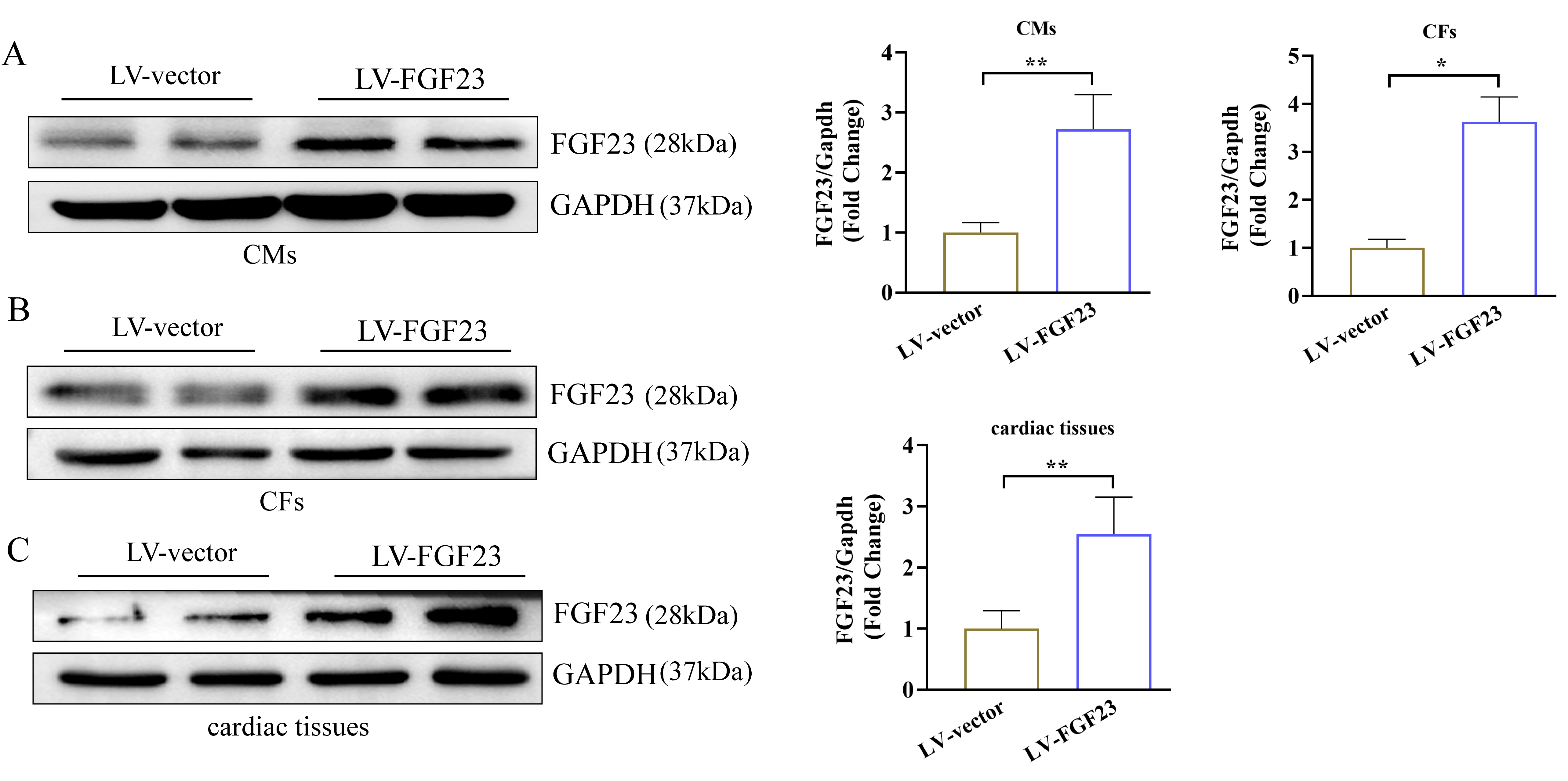

Supplement: Supplementary file 2 — Additional file 2: Figure S2. Verification of FGF23 overexpression. A–C, Immunoblots and quantification of FGF23 expression in CMs, CFs, and cardiac tissue.*P < 0.05 and **P < 0.01. [file 12967_2022_3482_MOESM2_ESM.jpg]
